# Bibliometric and visualized analysis of resveratrol in anticancer investigations

**DOI:** 10.1002/fsn3.3932

**Published:** 2024-01-06

**Authors:** Qiang Fu, Zhongqi Lu, Ying Chang, Tiefeng Jin, Meihua Zhang

**Affiliations:** ^1^ Department of Ultrasound Medicine Affiliated Hospital of Yanbian University Yanji P. R. China; ^2^ Department of Pathology and Cancer Research Center Yanbian University Medical College Yanji P. R. China; ^3^ Key Laboratory of the Science and Technology Department of Jilin Province Yanji P. R. China

**Keywords:** bibliometric analysis, cancer, hot research; research Progress, resveratrol, web of science

## Abstract

A growing number of publications have shown that resveratrol has anticancer effects and has become a hotspot in cancer research. The purpose of this study is to analyze the academic results and research trends in resveratrol within the field of anticancer and to predict the future trends in this field. We conducted a literature search for resveratrol in anticancer research from 2003 to 2022 using the Science Citation Index Expanded of the Web of Science Core Collection. The visualization software was used to perform the bibliometric analysis. A total of 1463 publications from 2003 to 2022 were retrieved. China had the highest number of publications. Taipei Medical University became the research institution with the largest number of publications worldwide. The journals with the highest output and co‐citation frequency were *Molecules* and *Cancer Research*. Levenson, Anait S and Jaeger, Walter published the largest number of papers. Jang, MS was the most co‐cited author. Timeline View shows trends and relationship between research topics over time and suggests that the emerging frontier of resveratrol in anticancer may be “resveratrol induces apoptosis.” As more and more evidence shows the important role of resveratrol in anticancer, further research on its mechanisms and target discovery may become a major direction for future research. The bibliometric analysis findings of this study will significantly contribute to scholars' comprehensive understanding of the anticancer effects and mechanisms of action of resveratrol, aiding in delineating research hotspots and frontier directions within this field, thereby providing guidance for future investigations.

## Graphical abstract

Resveratrol exerts its antitumor effects through multiple mechanisms and has great potential as an anticancer agent for the prevention and treatment of many types of cancer. The bibliometric analysis findings of this study will significantly contribute to scholars' comprehensive understanding of the anticancer effects and mechanisms of action of resveratrol, aiding in delineating research hotspots and frontier directions within this field, thereby providing guidance for future investigations.

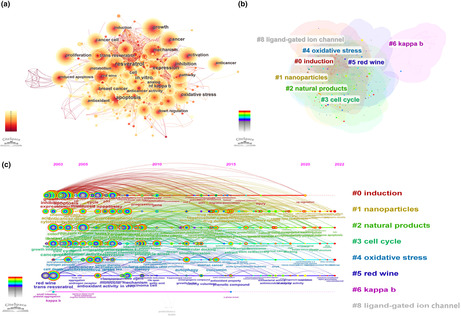

## 1 INTRODUCTION

As the global population ages, there is an escalating trend in the incidence and mortality rates of individuals afflicted with malignant neoplasms. Cancer, characterized by aberrant cellular proliferation, has emerged as a predominant public health challenge on a global scale (Bray et al., 2021). According to the GLOBOCAN 2020 report, the year 2020 witnessed 19,292,789 cases of cancer and 9,958,133 cancer‐related fatalities worldwide (Sung et al., 2021). The development of cancer unfolds as a protracted process during which normal cells accumulate genetic mutations, resulting in uncontrolled growth, infiltration, and metastasis to various organ systems, including but not limited to the liver, kidneys, spleen, lungs, intestines, and brain (Cao, 2019). The relentless objective of oncology researchers is to impede the advancement of cancer, forestall its metastatic spread, and enhance the quality of life for cancer patients.

In recent times, there has been a notable surge in scientific interest regarding natural product compounds due to their robust efficacy in mitigating diseases characterized by inflammation, notably cancer. A growing body of research substantiates the proposition that the incorporation of polyphenols into one's diet, prominently present in cereals, legumes, vegetables, and fruits, may serve as a prophylactic measure against the development of a spectrum of ailments, cancer included (Ko et al., 2017). Compounds capable of modulating these oncogenic mechanisms hold promise as prospective anticancer agents that could potentially progress to clinical utilization.

Resveratrol is a natural polyphenolic compound, abundant in various plant sources such as grapes, peanuts, blueberries, polygonum multiflorum, hairy quinoa rutabaga, cassia seeds, thuja, mulberry, and others. Its chemical structure is based on stilbene and consists of two phenolic rings linked by a styrene double bond, yielding 3,4,5‐trihydroxystilbene. This compound exists in two isoforms, trans‐ and cis‐, with the trans‐isoform being the predominant and extensively investigated form (Ko et al., 2017). Exposure to heat and ultraviolet radiation can induce the conversion of the trans‐isoform to the cis‐isoform, which structurally resembles the synthetic estrogen diethylstilbestrol, classifying resveratrol as a phytoestrogen. The biosynthesis of resveratrol initiates with the reaction between malonyl CoA and a coumaryl derivative, catalyzed by the enzyme stilbene synthase (Soleas et al., 1997). Resveratrol has been demonstrated as a promising environmentally friendly anticancer agent (Bishayee, 2009; Bishayee et al., 2010). It has also exhibited the capacity to reverse drug resistance in various tumor cell types by rendering them more responsive to chemotherapeutic agents (Lee et al., 2016, Mondal & Bennett, 2016). Resveratrol's anticancer effects are multifaceted, encompassing anti‐peroxidation, modulation of cellular redox metabolism, interference with autophagy, and reduction of cell invasiveness. As early as 1997, Jang et al. (1997) proposed its anticancer properties and elucidated potential mechanisms through in vitro experiments. Multiple studies have revealed its ability to hinder the proliferation of diverse tumor cells, including those found in colon, lung, prostate, bladder, breast, and thyroid cancers. Resveratrol can induce apoptosis in tumor cells through both extracellular and intracellular pathways, mediated by caspase‐8 and caspase‐9, respectively. Shimizu et al. (2006) observed resveratrol‐induced apoptosis in malignant B cells via the p38 MAP kinase signaling pathway. Furthermore, resveratrol exerts influence over the cell cycle in cancer cells, blocking the S phase and significantly inhibiting DNA synthesis (Heo et al., 2018). In the context of colorectal carcinogenesis, resveratrol plays an active role in the regulation of various factors, including interleukin‐6 (IL‐6), tumor protein p53, vascular endothelial growth factor (VEGF), and mitogen‐activated protein kinase 1 (Li et al., 2019).

In recent years, the compelling impact of resveratrol in restraining tumor cell proliferation through the induction of apoptosis and the inhibition of angiogenesis has garnered considerable attention from the research community. Resveratrol not only has the capacity to induce tumor cell apoptosis through intrinsic or extrinsic pathways and arrest the cell cycle but also exhibits the capability to hinder tumor migration by modulating signaling pathways associated with collagen degradation (Bishayee, 2009). Despite the substantial volume of resveratrol‐related anticancer literature published annually, researchers face a significant challenge in sifting through this extensive body of work, requiring them to assess the quality of articles and gauge their scientific impact and importance. In such circumstances, bibliometric analysis methods prove invaluable for evaluating articles across various disciplines (Zhang et al., 2022). This study aims to employ bibliometric methods to examine the trends and knowledge landscape in resveratrol‐related anticancer research over the past two decades. This paper offers an overview of this field, highlighting noteworthy research contributions and pinpointing emerging research areas.

## 2 MATERIALS AND METHODS

### 2.1 Data sources and search strategy

**FIGURE 1:**
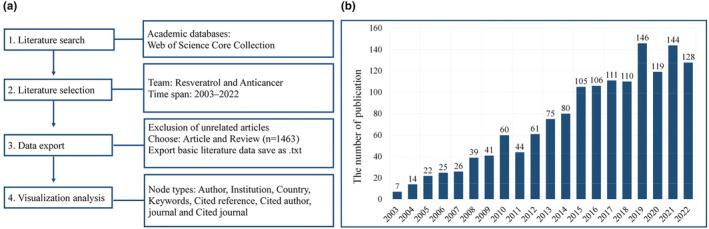
Publications related to resveratrol in anticancer. A flowchart of the publication screening process (a). Annual number of publications in this field (b).

A comprehensive literature search was conducted to retrieve publications pertaining to resveratrol and its role in anticancer research spanning from 2003 to 2022. The search was executed using the Web of Science Core Collection and was limited to articles and reviews. Importantly, no language restrictions were imposed. The search strategy employed the following query: “TS = (resveratrol) AND TS = (anticancer).” The Web of Science database offers access to valuable information such as the country of origin, institutional affiliations, author details, keywords, and reference lists for each publication, all of which are pertinent to this study. A total of 1463 articles were extracted from the Web of Science database in plain text format, while the full record and cited references in the document records were also obtained as source data for subsequent analysis. All retrieved records were collected as of November 30, 2022. A detailed outline of the retrieval strategy is depicted in Figure 1a.

### 2.2 Data analysis

All acquired documents were imported into three software applications: VOSviewer (version 1.6.15), CiteSpace (version 6.1.R6), and Microsoft Excel 2019. VOSviewer and CiteSpace are specialized tools designed for the creation and visualization of bibliometric networks, encompassing parameters such as countries, journals, and authors based on citation, co‐citation, and co‐authorship relationships. These software applications also enable the generation of co‐occurrence keyword visualizations to elucidate the knowledge structure within a given research domain and identify emerging trends (Chen, 2004; van Eck & Waltman, 2010). In the knowledge graph representations, individual nodes signify research items, with node size proportional to the frequency of mentions or citations. The connections between nodes denote co‐occurrences or co‐citations, and the thickness of these lines reflects the strength of the associations, with thicker lines indicating more robust relationships. Additionally, node and connection colors are indicative of the year of appearance (Chen, 2017). Detailed explanations of the methods for employing knowledge graph analysis software and the interpretation of these graphical representations can be found elsewhere (Chen et al., 2009, Lu et al., 2022). In this study, CiteSpace software was utilized to create knowledge maps that illustrate collaborative efforts between countries, institutions, and authors. It was also employed for co‐citation analysis of references, journal analysis for generating biplots, and examination of keywords with the development of timelines. Meanwhile, VOSviewer was utilized to construct network graphs for journal co‐occurrence and co‐citation, as well as keyword co‐occurrence.

## 3 RESULTS

### 3.1 Annual publication growth trends

A total of 1463 papers pertaining to resveratrol in the context of anticancer research from 2003 to 2022 were collected. Figure 1b presents the annual publication statistics for studies focusing on resveratrol and anticancer applications. In general, there has been a consistent increase in the number of publications over the years. During the initial phase (2003–2007), there was a relatively steady growth, with annual publication counts remaining below 40. However, a remarkable upswing in annual publication numbers commenced in 2019. In the past 4 years, the volume of published articles reached its zenith, exceeding 140 articles in the highest‐recorded year (*n* = 146). These findings underscore the mounting interest in research pertaining to resveratrol in anticancer, rendering it a prominent and rapidly evolving research area in recent years.

### 3.2 Country and institution analysis

**FIGURE 2:**
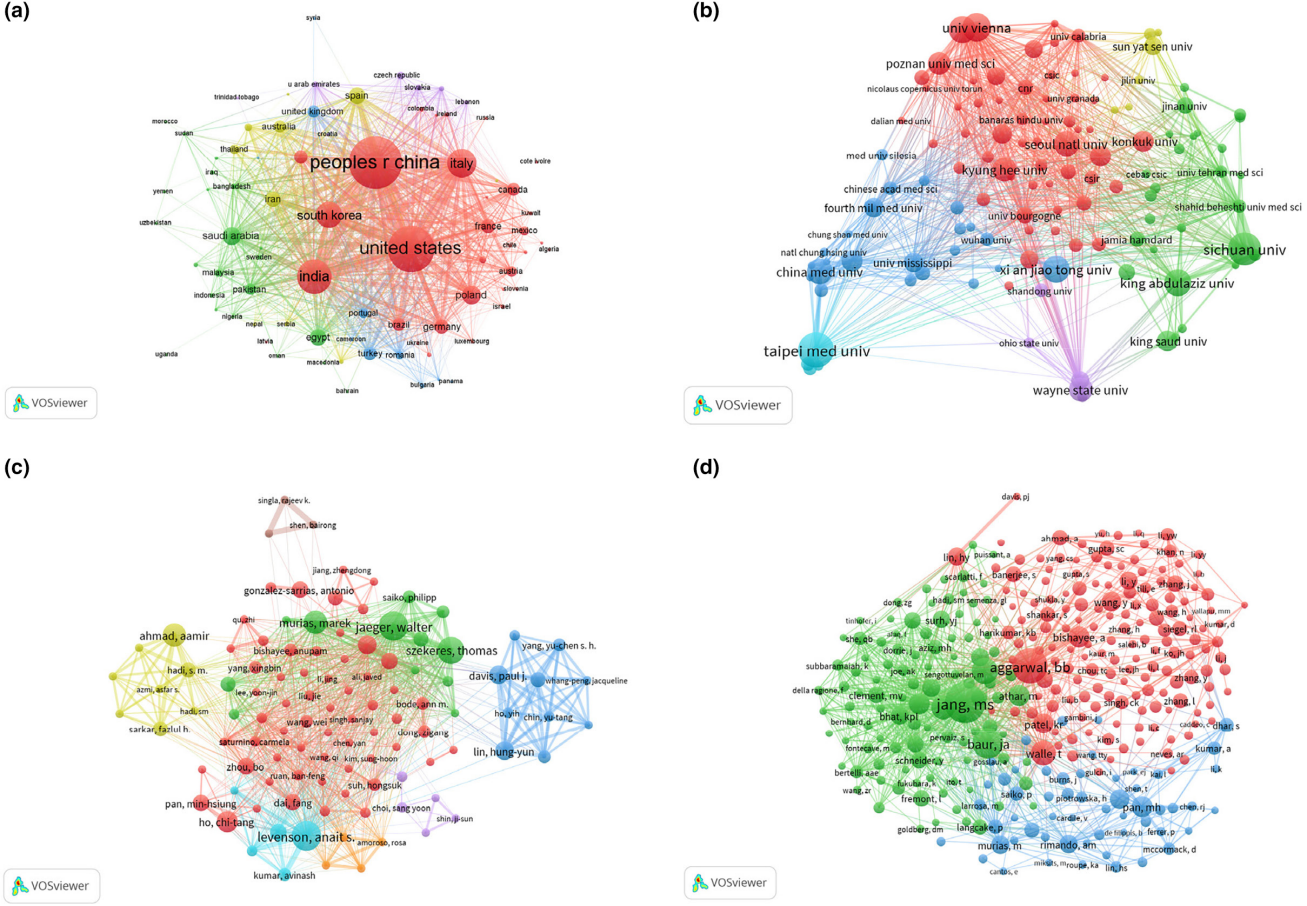
Network of countries(a), institutions(b), authors(c), and co‐cited authors(d) engaged in resveratrol in anticancer. In the network map (a or b), a node represents a country or institution. The larger the area of the node is, the larger the number of publications. The thicker the curved line connecting nodes indicates the frequency with which they co‐occur, as they indicate collaborative relationships. An isolated node without any connection is devoid of all collaboration. A node with a high betweenness centrality links two or more large groups of nodes. A node with a high betweenness centrality score exerts a strong influence on the network. In the network map (c or d), each node represents one author. Size of node is positively correlated with cited counts of the authors, and links between two circles represent a collaboration between two authors on the same article. Line thickness is positively correlated with frequency of collaborations.

**FIGURE 3:**
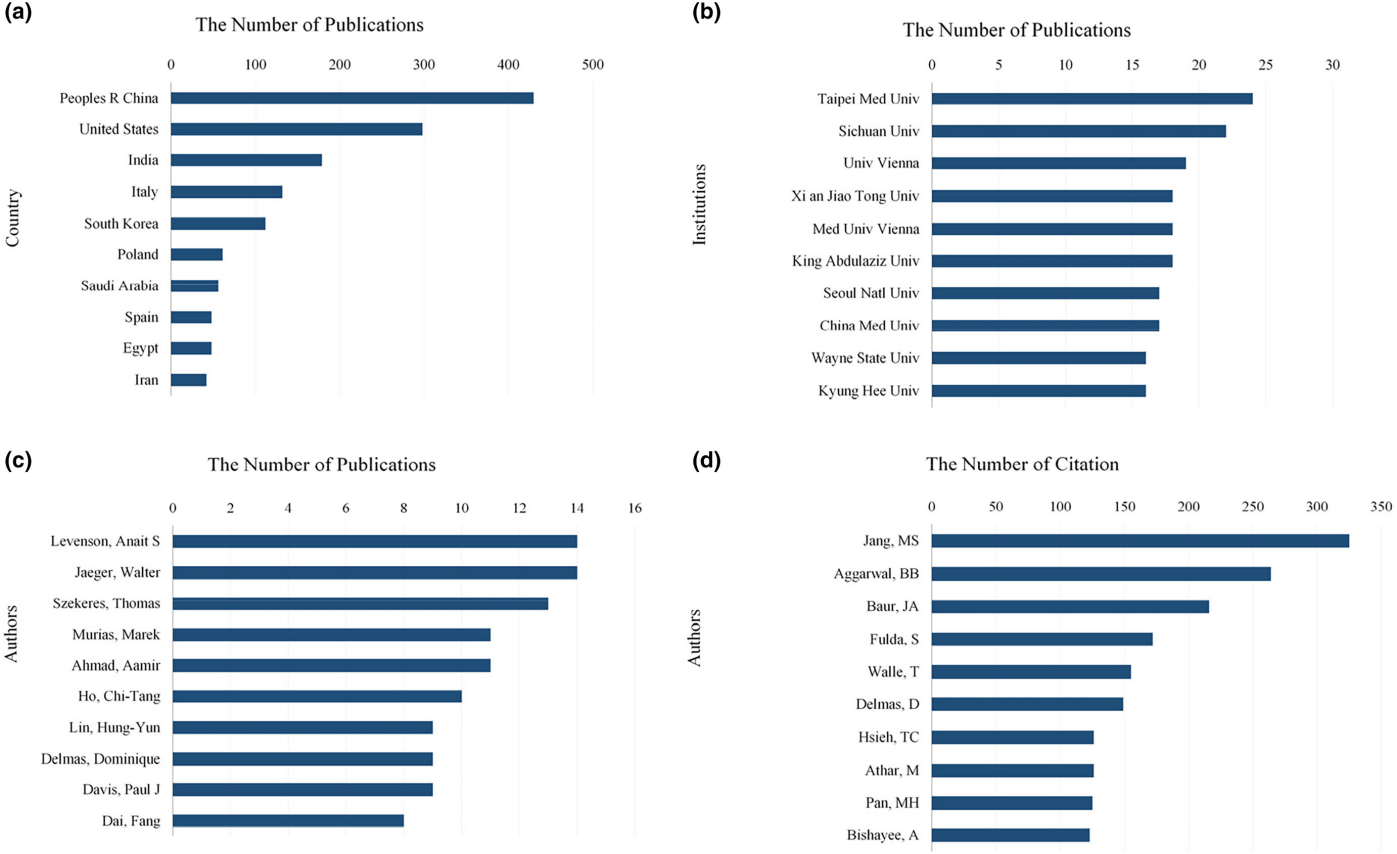
Publications and citation related to resveratrol in anticancer. The top 10 most productive countries (a). The top 10 most productive institutions (b). The number of author's publications (c). The number of author's citations (d).

A comprehensive examination of country collaborations and knowledge mapping to highlight key nations and their partnerships in the field was conducted using VOSviewer software (see Figure 2a). The dataset included a total of 1463 articles authored by 84 different countries. Figure 2a reveals extensive international cooperation, with China playing a prominent role in collaborations with numerous countries. The distribution of collaborative publications among the top 10 countries is illustrated in Figure 3a, with China, the United States, and India emerging as the top three contributors.

Figure 2b presents a visual representation of collaborative networks among research institutions within each country. This analysis encompassed contributions from 1891 institutions and depicted reciprocal collaborative relationships between these entities. The most significant global collaborations were observed with Chinese research institutions. Notably, four of the top 10 institutions were based in China. The top three institutions in terms of publication volume were Taipei Medical University, Sichuan University, and the University of Vienna (see Figure 3b). Chinese research institutions demonstrated close partnerships, both domestically and internationally, and led in terms of the volume of publications in this domain. This underscores their prominent role in global research related to resveratrol in anticancer applications, as they have made substantial contributions to the field.

### 3.3 Analysis of key authors and their collaborative networks

To discern the noteworthy authors in the field and explore their collaborative associations, we performed a statistical analysis using VOSviewer software (see Figure 2c). The collaborative network of authors illuminates the active participation of 7854 researchers in resveratrol‐related anticancer research. As depicted in Figure 3c, the top three authors with the most published papers are Levenson, Anait S (*n* = 14), Jaeger, Walter (*n* = 14), and Szekeres, Thomas (*n* = 13). As depicted by the collaboration network of the authors in Figure 2c, five major collaborative networks have emerged: the light blue network (centered around Levenson, Anait S), the green network (centered around Jaeger, Walter, Szekeres, Thomas, and Murias, Marek), the red network (centered around Ho, Chi‐tang), and the dark blue network (centered around Lin, Hung‐Yun and Davis, Paul J). These five networks demonstrate not only strong internal collaborative relationships but also close collaborations among themselves, reflecting extensive international collaborative efforts.

Author co‐citation analysis, a valuable tool for identifying research trends within resveratrol's application in anticancer research, reveals co‐citation relationships formed when two or more authors are simultaneously cited in subsequent papers. As depicted in Figure 2d, a closely interconnected network of authors emerges, indicating similarity in their research domains and mutual citations. Among the top 10 co‐cited authors (Figure 3d), Jang, MS (*n* = 325) holds the foremost position, followed by Aggarwal, BB (*n* = 264) and Baur, JA (*n* = 216). A discernible co‐occurrence relationship between authors and co‐cited authors is observed, with authors who have a higher publication count tending to exhibit increased co‐occurrence rates with other authors. Jang, MS, in particular, exerts a substantial influence in the realm of resveratrol's application in anticancer research, as evidenced by extensive citations of his/her work by fellow researchers, with a remarkable co‐citation frequency of 325 instances.

### 3.4 Journals and co‐cited scholarly journals

**FIGURE 4:**
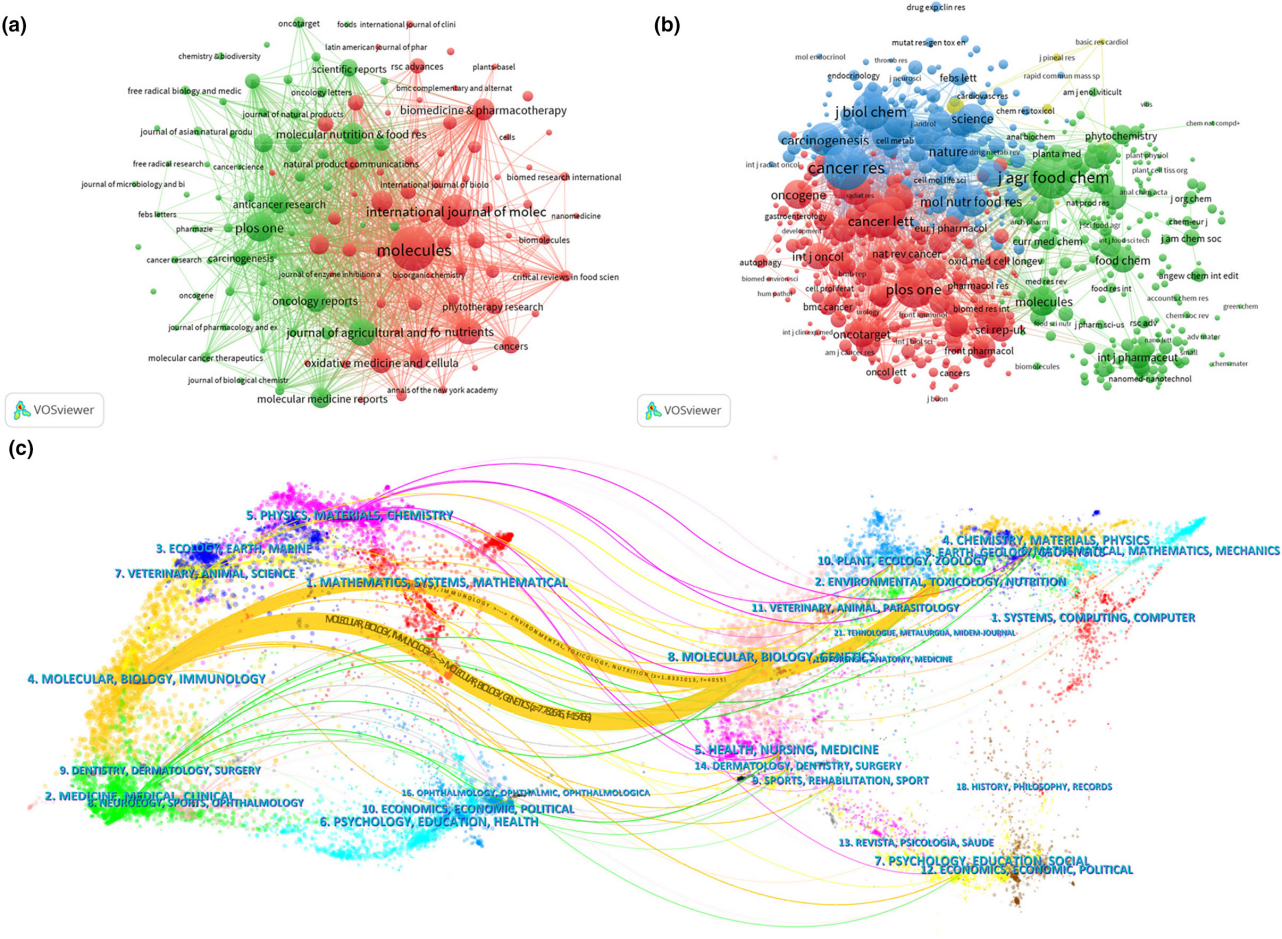
The network map of scholarly journals (a). The network map of co‐cited scholarly journals (b). A dual‐map overlay of journals related to research on resveratrol in anticancer (c).

A total of 564 academic journals have published research papers on resveratrol in the context of anticancer investigations (see Figure 4a). Notably, *Molecules* (*n* = 50) emerged as the most prolific publisher, followed by the *International Journal of Molecular Sciences* (*n* = 32) and *PLoS One* (*n* = 27). Significantly, seventy percent (70%) of the journals boasted an Impact Factor (IF) exceeding 5.0 in 2022, with six of them (60%) positioned in the JCR (2022) Q1 region. *Molecules* stood out with the highest total citations, accumulating 14,068 citations (Table 1). Of the 7289 co‐cited academic journals, *Cancer Research* garnered more than 2500 citations. Among the top 10 co‐cited journals, *Nature* held the highest IF (IF = 69.5), followed by *Cancer Research* (IF = 13.3) and *Cancer Letters* (IF = 9.7) (Table 2). As depicted in Figure 4a, a positive citation relationship is discernible among various journals.

**TABLE 1:** Top 10 most published journals related to resveratrol in anticancer.

Rank	Journal	Documents	Total link strength	JCR category quartile	IF (2021)
1	*Molecules*	50	14,068	Biochemistry, Genetics, and Molecular Biology (Q2)	4.9
2	*International Journal of Molecular Sciences*	32	7227	Biochemistry, Genetics, and Molecular Biology (Q1)	6.2
3	*Plos One*	27	7694	Multidisciplinary (Q2)	3.7
4	*Journal of Agricultural and Food Chemistry*	24	6903	Agricultural and Biological Sciences (Q1)	5.8
5	*Nutrients*	23	8453	Agricultural and Biological Sciences (Q1)	6.7
6	*Oncology Reports*	19	4457	Biochemistry, Genetics, and Molecular Biology (Q3)	4.1
7	*Biomedicine & Pharmacotherapy*	18	5650	Medicine (miscellaneous) (Q1)	7.4
8	*Molecular Nutrition & Food Research*	18	5830	Agricultural and Biological Sciences (Q1)	6.5
9	*European Journal of Medicinal Chemistry*	17	3589	Chemistry (Q1)	7
10	*Oxidative Medicine and Cellular Longevity*	17	5794	Biochemistry, Genetics, and Molecular Biology (Q2)	7.3

**TABLE 2:** Top 10 most co‐cited journals related to resveratrol in anticancer.

Rank	Co‐cited journals	Citations	Total link strength	JCR category quartile	IF (2021)
1	*Cancer Research*	2113	197,913	Biochemistry, Genetics, and Molecular Biology (Q1)	13.3
2	*Journal of Agricultural and Food Chemistry*	1974	170,744	Agricultural and Biological Sciences(Q1)	5.8
3	*PLoS One*	1475	153,143	Multidisciplinary (Q2)	3.7
4	*Journal of Biological Chemistry*	1367	121,961	Biochemistry, Genetics, and Molecular Biology (Q2)	5.4
5	*Cancer Letters*	1211	126,155	Biochemistry, Genetics, and Molecular Biology (Q1)	9.7
6	*Carcinogenesis*	1068	113,975	Biochemistry, Genetics, and Molecular Biology (Q2)	4.7
7	*Molecular Nutrition and Food Research*	997	89,205	Agricultural and Biological Sciences (Q1)	6.5
8	*Biochemical Pharmacology*	956	100,129	Biochemistry, Genetics, and Molecular Biology(Q1)	6.1
9	*Nature*	911	81,207	Multidisciplinary (Q1)	69.5
10	*International Journal of Cancer*	874	90,489	Biochemistry, Genetics, and Molecular Biology (Q1)	7.3

To construct citation network graphs, journals with issue numbers greater than or equal to 3 (*T* = 3) were employed. Co‐citation network construction relied on journals with a co‐citation count equal to or exceeding 30 (*T* = 30), as presented in Figure 4b.

Figure 4c, the dual‐map of journals, depicts the thematic distribution of these journals. The citing journal is situated on the left side of the map, while the cited journal is positioned on the right. The tags on the map represent the academic disciplines covered by the respective journals. Colored lines traverse from left to right, delineating reference pathways. A primary citation pathway is evident, and the yellow pathway signifies that journals specializing in *Molecular/Biology/Genetics and Environmental/Toxicology/Nutritio*n frequently cite studies from journals focusing on *Molecular/Biology/Immunology*.

### 3.5 Analysis of co‐cited references and references burst

**FIGURE 5:**
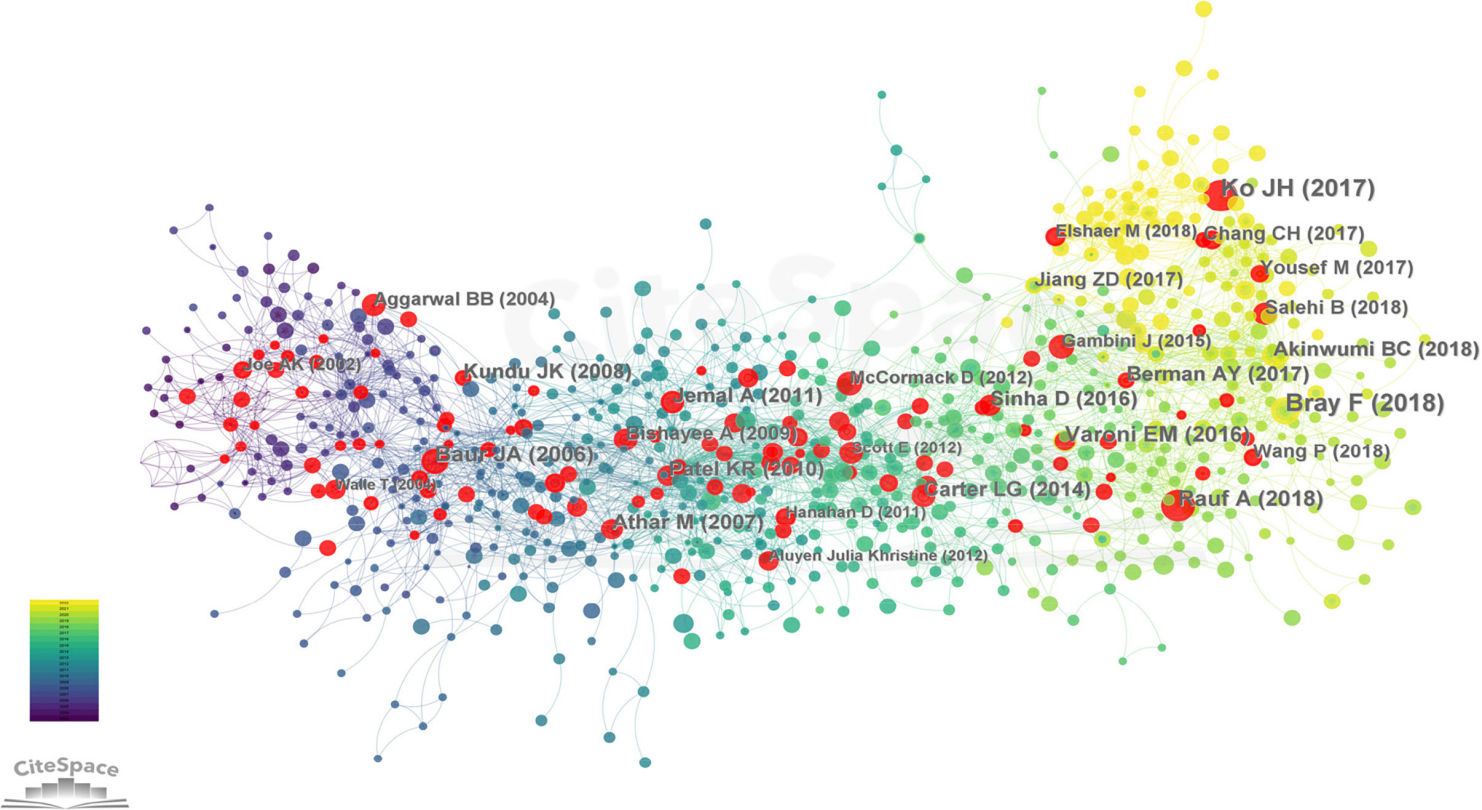
A network map showing co‐cited references involved in the research on resveratrol in anticancer. Each node represents a reference. Size of node is positively correlated with frequency of citations, and links between two circles represent two references that were cited in the same article (red nodes represent references with the strongest citation bursts).

A co‐citation network was employed to investigate the pivotal references in the realm of resveratrol's application in anticancer research. In this network, nodes were designated to represent references, specifically citations, to construct a co‐citation network of the literature (see Figure 5). The ten most frequently cited references are detailed in Table 3 (Aggarwal et al., 2004; Athar et al., 2007; Baur & Sinclair, 2006; Boocock et al., 2007; Clément et al., 1998; Frémont, 2000; Jang et al., 1997; Kundu & Surh, 2008; Patel et al., 2010; Walle et al., 2004). Among the top 10 references, four (40%) have garnered citations exceeding 100 times. The paper titled “Cancer chemopreventive activity of resveratrol, a natural product derived from grapes,” authored by Jang MS et al. and published in 1997, holds the highest number of co‐citations (*n* = 314). Furthermore, it boasts an impressive IF for 2022, standing at 63.7, and is classified within the Q1 category (Jang et al., 1997). Furthermore, the paper titled “Therapeutic potential of resveratrol: the in vivo evidence,” authored by Baur JA et al. and published in 2006, has secured the highest IF (IF = 112.2) among the references, accompanied by 152 citations (Baur & Sinclair, 2006).

**TABLE 3:** Top 10 citations related to resveratrol in anticancer.

Co‐cited reference	Count	Title	IF	JCR
Jang MS, 1997, *Science*, v275, p218, doi 10.1126/science.275.5297.218	314	Cancer chemopreventive activity of resveratrol, a natural product derived from grapes	63.7	Q1
Baur JA, 2006, *Nat Rev Drug Discov*, v5, p493, doi 10.1038/nrd2060	152	Therapeutic potential of resveratrol: the in vivo evidence	112.2	Q1
Aggarwal BB, 2004, *Anticancer Res*, v24, p2783	142	Role of resveratrol in prevention and therapy of cancer: preclinical and clinical studies	2.4	Q4
Walle T, 2004, *Drug Metab Dispos*, v32, p1377, doi 10.1124/dmd.104.000885	108	High absorption but very low bioavailability of oral resveratrol in humans	3.5	Q2
Clement MV, 1998, *Blood*, v92, p996	80	Chemopreventive agent resveratrol, a natural product derived from grapes, triggers CD95 signaling‐dependent apoptosis in human tumor cells	25.4	Q1
Athar M, 2007, *Toxicol Appl Pharm*, v224, p274, doi 10.1016/j.taap.2006.12.025	78	Resveratrol: a review of preclinical studies for human cancer prevention	4.4	Q2
Fremont L, 2000, *Life Sci*, v66, p663, doi 10.1016/s0024‐3205(99)00410‐5	73	Biological effects of resveratrol	6.7	Q1
Patel KR, 2010, *Cancer Res*, v70, p7392, doi 10.1158/0008‐5472.can‐10‐2027	66	Clinical pharmacology of resveratrol and its metabolites in colorectal cancer patients	13.3	Q1
Kundu JK, 2008, *Cancer Lett*, v269, p243, doi 10.1016/j.canlet.2008.03.057	64	Cancer chemopreventive and therapeutic potential of resveratrol: mechanistic perspectives	9.7	Q1
Boocock DJ, 2007, *Cancer Epidem Biomar*, v16, p1246, doi 10.1158/1055‐9965.epi‐07‐0022	59	Phase I dose escalation pharmacokinetic study in healthy volunteers of resveratrol, a potential cancer chemopreventive agent	4	Q2

The identification of the top 20 references with the most pronounced citation bursts was accomplished through an analysis of literature co‐citation intensity using CiteSpace software, a technique employed for detecting prevailing research trends. In Figure 5, references with the most prominent citation bursts are denoted by red nodes. A “citation burst” signifies that a particular reference has received widespread citations over an extended timeframe. In Table 4, periods highlighted in red signify a sudden surge in citation frequency during that period, while blue designates a phase of relatively lower popularity. As illustrated in Table 4, among the 20 references with the Strongest Citation Bursts (Aggarwal et al., 2004; Athar et al., 2007; Baur et al., 2006; Baur & Sinclair, 2006; Berman et al., 2017; Bishayee, 2009; Boocock et al., 2007; Brown et al., 2010; Carter et al., 2014; Gusman et al., 2001; Jemal et al., 2011; Ko et al., 2017; Kundu & Surh, 2008; Patel et al., 2010; Rauf et al., 2018; Saiko et al., 2008; Salehi et al., 2018; Sinha et al., 2016; Varoni et al., 2016; Wang & Sang, 2018). Wang and Sang (2018), Salehi et al. (2018), Rauf et al. (2018), and Ko et al. (2017) are burst references in 2019–2022, with burst strengths of 7.91, 7.91, 11.72, and 18.26. It is foreseeable that the research directions delineated in these three articles will likely become prominent focal points in the coming years and will garner increased citations from scholars. Citation (Ko et al., 2017) has the Strongest Citation Bursts (strength = 18.26) and lasts for 4 years (2019–2022). In other words, it is evident that the articles authored by Ko JH et al. have received a substantial number of citations. The citation (Sinha et al., 2016) (strength = 8.98) bursts from 2017 to 2022, and the citation (Berman et al., 2017) (strength = 7.65) bursts from 2018 to 2022.

**TABLE 4:** Top 20 references with the strongest citation bursts.

References	Strength	Begin	End	2003–2022
Gusman J, 2001, *Carcinogenesis*, V22, P1111, doi 10.1093/carcin/22.8.1111	7.64	2003	2006	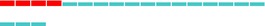
Aggarwal BB, 2004, *Anticancer Res*, V24, P2783	9.87	2006	2009	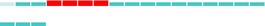
Baur JA, 2006, *Nat Rev Drug Discov*, V5, P493, doi 10.1038/nrd2060	15.91	2007	2011	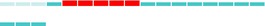
Baur JA, 2006, *Nature*, V444, P337, doi 10.1038/nature05354	9.4	2007	2011	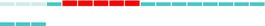
Athar M, 2007, *Toxicol Appl Pharm*, V224, P274, doi 10.1016/j.taap.2006.12.025	13.67	2008	2012	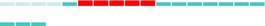
Boocock DJ, 2007, *Cancer Epidem Biomar*, V16, P1246, doi 10.1158/1055‐9965.EPI‐07‐0022	9.24	2008	2012	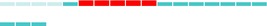
Kundu JK, 2008, *Cancer Lett*, V269, P243, doi 10.1016/j.canlet.2008.03.057	10.25	2009	2013	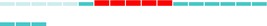
Saiko P, 2008, *Mutat Res‐Rev Mutat*, V658, P68, doi 10.1016/j.mrrev.2007.08.004	8.38	2009	2013	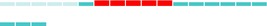
Bishayee A, 2009, *Cancer Prev Res*, V2, P409, doi 10.1158/1940‐6207.CAPR‐08‐0160	10.07	2010	2013	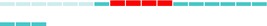
Patel KR, 2010, *Cancer Res*, V70, P7392, doi 10.1158/0008‐5472.CAN‐10‐2027	10.77	2011	2015	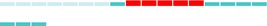
Brown VA, 2010, *Cancer Res*, V70, P9003, doi 10.1158/0008‐5472.CAN‐10‐2364	8.06	2011	2015	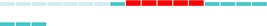
Jemal A, 2011, *CA‐Cancer J Clin*, V61, P69, doi 10.3322/caac.20107, 10.3322/caac.20115	10.22	2013	2016	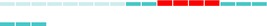
Carter LG, 2014, *Endocr‐Relat Cancer*, V21, PR209, doi 10.1530/ERC‐13‐0171	10.21	2016	2019	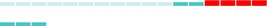
Varoni EM, 2016, *Front Nutr*, V3, P0, doi 10.3389/fnut.2016.00008	11.91	2017	2019	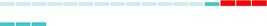
Sinha D, 2016, *Semin Cancer Biol*, V40‐41, P209, doi 10.1016/j.semcancer.2015.11.001	8.98	2017	2022	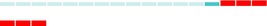
Berman AY, 2017, *NPJ Precis Oncol*, V1, P0, doi 10.1038/s41698‐017‐0038‐6	7.65	2018	2022	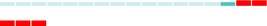
Ko JH, 2017, *Int J Mol Sci*, V18, P0, doi 10.3390/ijms18122589	18.26	2019	2022	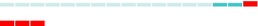
Rauf A, 2018, *Crit Rev Food Sci*, V58, P1428, doi 10.1080/10408398.2016.1263597	11.72	2019	2022	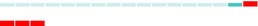
Salehi B, 2018, *Biomedicines*, V6, P0, doi 10.3390/biomedicines6030091	7.91	2019	2022	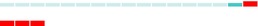
Wang P, 2018, *Biofactors*, V44, P16, doi 10.1002/biof.1410	7.91	2019	2022	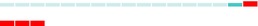

### 3.6 Analysis of keywords and keyword co‐occurrence cluster

**FIGURE 6:**
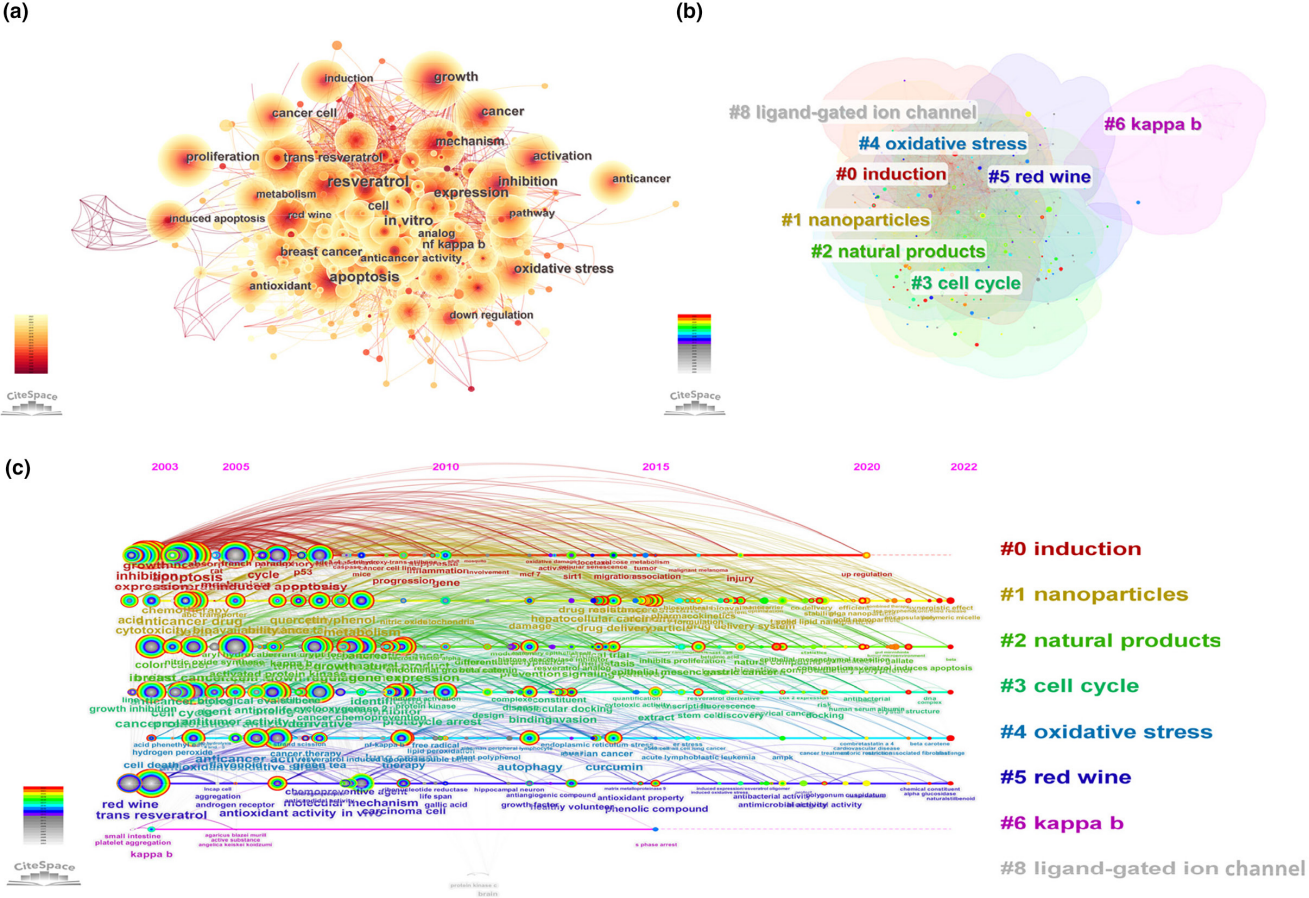
A network map of keywords involved in the research on resveratrol in anticancer. Co‐occurrence analysis of keywords (a). Cluster networks of keywords via CiteSpace (b). Timeline view of keywords involved in resveratrol in anticancer (c).

Keywords serve as a concise summary of research themes within the literature. Analyzing the co‐occurrence of keywords allows for an insight into the evolution of prominent research areas within a specific field. To delve deeper into the examination of research trends in the context of resveratrol's application in anticancer research, we established a keyword co‐occurrence network (see Figure 6a) (density = 0.0382, N = 499, E = 4749). Table 5 gives the keywords with higher frequency that have appeared in the past two decades, which can represent the main focus directions of resveratrol in anticancers. The keywords of the hotspot include resveratrol, apoptosis, anticancer, inhibition, trans‐resveratrol, oxidative stress, activation, curcumin, NF‐kB signaling, induced apoptosis, anticancer activity, etc. The high frequency of resveratrol, apoptosis, anticancer, induced apoptosis, and anticancer activity indicates that resveratrol is getting more and more attention in the field of anticancer.

**TABLE 5:** Top 20 keywords related to the field of resveratrol in anticancer.

Rank	Keyword	Occurrences	Rank	Keyword	Occurrences
1	Resveratrol	414	11	Activation	122
2	Apoptosis	299	12	Cell	121
3	In vitro	253	13	Mechanism	118
4	Expression	201	14	NF kappa b	108
5	Cancer	154	15	Cancer cell	104
6	Inhibition	153	16	Proliferation	96
7	Growth	147	17	Anticancer	80
8	Oxidative stress	127	18	Pathway	79
9	Trans‐resveratrol	125	19	Antioxidant	77
10	Breast cancer	123	20	Anticancer activity	69

Clustering analysis is a statistical method for classifying data with multiple indicators. We conducted a cluster network analysis based on the similarity between keywords. With the direction of research related to resveratrol in anticancer, clustering was performed on the basis of keyword co‐occurrence analysis, and a total of eight clusters were formed (Figure 6b), density = 0.0382, N = 499, E = 4749, and Weighted Mean Silhouette = 0.6649 (range 0 to 1), which indicates that the clustering results can reflect the real situation. From Figure 6c, it can be clearly seen that the research directions related to resveratrol in anticancer are mainly concentrated in eight aspects: according to the frequency from high to low: #0 induction, #1 natural products, #2 nanoparticles, #3 cell cycle, #4 oxidative stress, #5 red wine, #6 kappa b, and #8 ligand‐gated ion channel.

### 3.7 Analysis of hotspots and frontiers

To investigate the progression of research topics, we utilized CiteSpace software to create timeline graphs, with keywords represented as nodes (see Figure 6c) (Wang et al., 2021), density = 0.0382, N = 499, E = 4749. The timeline view is a data visualization approach that combines clustering and temporal segmentation methods to illustrate the evolution of research topics and their interconnections over time. Clustering labels are organized based on their chronological emergence. These labels not only provide insights into the distribution of research subjects but also convey the evolution of research hotspots across time. In the timeline view, colors within nodes on the same row represent different years, with older keywords positioned to the left and the most recent keywords to the right. Keywords sharing the same horizontal line belong to the same cluster, and the summarized labels are situated at the far right end of the row. Figure 6c offers a clear depiction of trends and relationships among research themes across time and suggests that emerging frontiers in the field of resveratrol in anticancer research encompass topics such as “resveratrol induces apoptosis,” “tumor microenvironment,” and “synergistic effect.”

Keywords exhibiting the Strongest Citation Bursts serve not only as indicators of prevalent research areas within a specific timeframe but also as prognosticators of forthcoming trends in the field over recent years. In this context, the presence of a red color signifies a sudden surge in the utilization of a keyword during the indicated period, while a blue line denotes a period of relatively diminished popularity. As shown in Table 6, “red wine” has the Strongest Citation Bursts (strength = 13.03, 2003–2010), followed by “grape” (strength = 8.37, 2003–2009), which represents the richer content of resveratrol in red wine and grape. The bursts words such as “combination,” “natural product,” “cell cycle arrest,” and “upregulation” are the key words that burst during 2020–2022, which represents the ability of resveratrol to inhibit tumor cell proliferation through rational cell cycle arrest. The bursts word “colorectal cancer” is the key word that burst during 2021–2022, which represents a significant inhibitory effect of resveratrol on the invasion and migration ability of colon cancer cells and colorectal cancer treatment will be the hotspots for resveratrol research within the oncology field.

**TABLE 6:** Top 25 keywords with the strongest citation bursts.

Keywords	Strength	Begin	End	2003–2022
Red wine	13.03	2003	2010	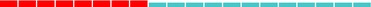
Grape	8.37	2003	2009	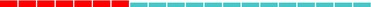
Wine	7.92	2004	2013	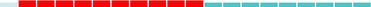
Chemopreventive agent resveratrol	5.15	2004	2011	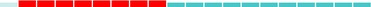
Inhibition	4.39	2004	2006	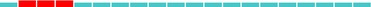
Nitric oxide synthase	5.82	2005	2012	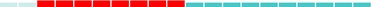
Trans‐resveratrol	4.43	2005	2009	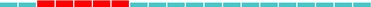
Induced apoptosis	5.46	2006	2014	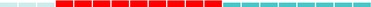
Phosphorylation	4.78	2007	2014	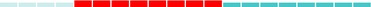
Breast cancer cell	4.94	2008	2014	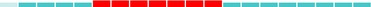
Analog	4.78	2009	2013	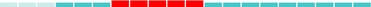
Carcinoma cell	4.21	2009	2014	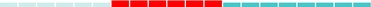
Apoptosis	5.1	2011	2011	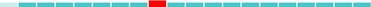
Modulation	4.75	2012	2013	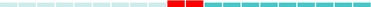
Cycle	4.16	2012	2015	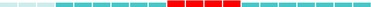
Multidrug resistance	6.22	2016	2018	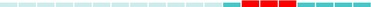
Design	4.24	2017	2020	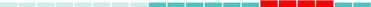
Target	5.61	2018	2020	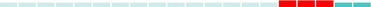
Curcumin	5.29	2019	2022	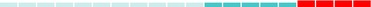
Solid lipid nanoparticle	5.09	2019	2020	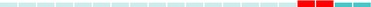
Combination	5.4	2020	2022	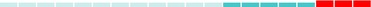
Natural product	4.94	2020	2022	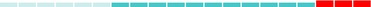
Cell cycle arrest	4.73	2020	2022	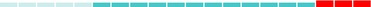
Upregulation	4.23	2020	2022	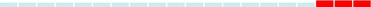
Colorectal cancer	4.51	2021	2022	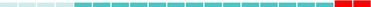

## 4 DISCUSSION

### 4.1 General information and research hotspots

This study elucidates the research trajectory of resveratrol in anticancer applications spanning from 2003 to 2022. It offers a comprehensive overview of its historical development and provides a more precise and dependable foundation for forecasting future trends. The analysis results demonstrate an exponential growth in the annual number of publications over the past two decades, with annual publication counts surging from 7 to over 140. Building upon this growth pattern, it is anticipated that this upward trend will persist in the coming years. Among countries, China exhibited the highest publication output, with Taipei Medical University emerging as the leading research institution worldwide. Molecules and Cancer Research claimed the top positions for journal productivity and co‐citation frequency, respectively. In terms of individual authors, Levenson, Anait S, and Jaeger, Walter held the record for the most published papers, while Jang, MS emerged as the most frequently co‐cited author in the field.

Keywords are a high summary of the research themes and core contents of the literature. All keywords were classified into eight clusters, including induction, natural products, nanoparticles, cell cycle, oxidative stress, red wine, kappa b, and ligand‐gated ion channel. High‐frequency keywords include resveratrol, apoptosis, anticancer, inhibition, trans‐resveratrol, oxidative stress, activation, curcumin, NF‐kB signaling, induced apoptosis, etc. Recent keywords with high burst strength include combination, natural product, cell cycle arrest, and upregulation. Timeline View shows trends and relationship between research themes over time and suggests the newly emerging frontier of resveratrol in anticancer may be “resveratrol induces apoptosis,” “tumor microenvironment,” and “synergistic effect.” In short, the latest trend of resveratrol in anticancer research is that resveratrol, as a natural anticancer substance, has strong inhibitory activity against many types of tumors in vitro and in vivo. It can play a role in cancer treatment and prevention by regulating cell cycle, modulating oncogenes, blocking cancer cell pathways, inducing cell autophagy, and inducing apoptosis. Further research on the molecular mechanisms of anticancer and targeted cancer therapy is needed to improve anticancer efficacy.

### 4.2 Resveratrol in anticancer

Polyphenols, alkaloids, and terpenoids exhibit promising therapeutic potential in addressing conditions such as cancer, oxidative stress, inflammation, ulcers, diabetes, platelet aggregation, microbial resistance, and tumors (Riaz et al., 2023). Stilbenes are considered important metabolically active polyphenols (Shazmeen et al., 2021). Stilbenes represent a commonly encountered structural scaffold in nature, and extensive research has been conducted on the biological functionalities of derivatives of trans‐stilbenes. Notably, resveratrol and its naturally occurring trans‐stilbene derivatives have been extensively investigated for their antioxidative, anti‐inflammatory, and anticancer properties (Li et al., 2023). Resveratrol is a natural polyphenol compound found in various plants, and it can be derived from over 70 plant sources (Aggarwal et al., 2004; Wang, Li, et al., 2023). Substantial research findings consistently underscore its inhibitory effect on cancer development (Ko et al., 2017). Resveratrol functions as a chemopreventive agent in all four key phases of carcinogenesis, encompassing initiation, promotion, progression, and metastasis (Jang et al., 1997). Moreover, it has exhibited effectiveness in both in vitro and in vivo cancer treatment settings (Baek et al., 2016). Resveratrol's mechanisms of action include the inhibition of proliferation in numerous cancer cell types (Wang, Gao, et al., 2023), such as lymphoma, multiple myeloma, as well as breast, prostate, stomach, colon, pancreatic, thyroid, head and neck squamous cell, ovarian, and cervical cancers.

### 4.3 Resveratrol regulates the cancer cell cycle

ATMACA H et al. showed that resveratrol inhibited the proliferation of PC‐3 cells, blocked prostate cancer (PCa) PC‐3 cells in G0/G1 phase, upregulated Bax protein expression, downregulated Survivin protein expression, and inhibited PC‐3 cell proliferation by directly activating Caspase‐3 (Atmaca et al., 2016). Resveratrol also plays an important role in the proliferation and migration of PCa cells. It acts as a target for lysosomal degradation, inhibits TNF‐receptor‐associated factor 6, a mediator of PCa cell proliferation and migration (Khusbu et al., 2020), slows PC‐3 cell growth by interfering with glucose fermentation and promoting respiration, and effectively inhibits PC‐3 cell growth under hypoxic conditions (Fonseca et al., 2019).

### 4.4 Resveratrol induces tumor cell apoptosis

Apoptosis is a genetically regulated, autonomous, and highly organized process by which cells undergo programmed cell death to preserve the stability of the internal cellular environment. This process involves two primary signal transduction pathways based on the source of apoptotic signals: the extrinsic pathway (also known as the death receptor pathway) and the intrinsic pathway (often referred to as the mitochondrial pathway). Ultimately, both pathways converge to activate downstream effector caspases. miRNAs are a potential method for the diagnosis and treatment of breast cancer, and are involved in cell cycle regulation and are associated with proliferation and apoptosis. Resveratrol can promote apoptosis by controlling a series of miRNAs in breast cancer cells (Kong et al., 2014; Venkatadri et al., 2016). Threonine kinase (Akt) is a transactivating signal of PI3K that inhibits apoptosis. Jehan et al. (Hussain et al., 2011) showed that resveratrol induces inactivation of the Akt pathway by initiating ROS, which leads to apoptosis of cancer cells. Song et al.'s research indicates that resveratrol can decrease the expression of Bax and the activity of Caspase‐3, while simultaneously increasing the expression of Bcl‐2 and the activity of SIRT1 (Song et al., 2023).

### 4.5 Resveratrol regulates redox metabolism in tumor cells

Oxidative stress refers to a state of imbalance in the production and consumption of reactive oxygen species (ROS) in cells, and oxidative stress causes apoptosis mainly through the mitochondrial and non‐mitochondrial pathways. Most tumor cells exhibit increased aerobic glycolysis (Warburg effect) and high oxidative stress than normal cells. High oxidative stress tumor cells are more susceptible to damage by exogenous ROS inducers, so modulation of ROS levels can be used as a method to kill tumor cells (Pavlova & Thompson, 2016). Juan et al. (2008) found that trans‐resveratrol could induce apoptosis in colon cancer HT‐29 cells by activating caspase‐3 through the production of superoxide radicals. It was also found that low doses of resveratrol inhibited the proliferation of non‐small cell lung cancers A549 and H460 mainly through a non‐mitochondrial pathway, activating NAPDH oxidase (NOX‐5) to produce ROS and inducing upregulation of p53 and p21 genes, which in turn affected cellular DNA damage (Luo et al., 2013). Lucas and Kolodziej (2015) found that 65 μmol/L of trans‐resveratrol on lung cancer A549 cells induced ROS production and stimulated caspase‐3 production, but caspase‐8 did not change, suggesting that ROS stimulation was the mitochondrial pathway to induce apoptosis.

### 4.6 Resveratrol inhibits cancer metastasis

PCa is acknowledged to be influenced by dihydrotestosterone acting through the androgen receptor (AR). In the context of cancer metastasis, the chemokine receptor CXCR4 has been observed to undergo upregulation and has been employed as a prognostic indicator in diverse cancer types, including leukemia, breast cancer, and PCa. Jang et al. (2019) demonstrated that resveratrol combined with AR and CXCR4 antagonists can be used to inhibit metastasis of PCa. Tsai et al. (2019) investigated the effect of resveratrol on neutrophil function and signaling mechanism. Resveratrol inhibited the phosphorylation of Src family kinases (SFKs) and decreased their enzymatic activity. In addition, resveratrol and the selective inhibitor SFKs (PP2) decreased the phosphorylation of tyrosine kinases and Vav in Bruton, suggesting that the inhibitory effect of resveratrol is achieved by inhibiting the SFKs‐Btk‐Vav pathway.

### 4.7 Resveratrol blocks cancer cell pathway

The excessive response to abnormal signals regulating growth hormone can promote tumor formation. In a study of head and neck squamous cell carcinoma, Baek et al. (2016) showed that resveratrol inhibited the proliferation of cancer cells by blocking the STAT3 signaling pathway through the induction of cytokine signal transduction protein 1, and the cell cycle was blocked in the G0 phase. In a recent study, Yang et al. (2016) concluded that the expression of tumor necrosis factor‐β1 (TGF‐β1) and Smad4 was significantly reduced by resveratrol in human rhabdomyosarcoma cells, and this inhibition was time‐ and concentration‐dependent, and its anticancer properties were produced by inhibiting the activation of the TGF‐β1/Smad signaling pathway. Ji et al. (2013) showed that Res inhibited the expression of c‐Myc, matrix metalloproteinase‐7 (MMP‐7), and lung adenocarcinoma metastasis‐associated transcript 1 (MALAT1) by suppressing the expression of target genes of Wnt/β‐catenin signaling pathway. Roy et al. (2009) found that the killing effect of Res‐promoted UV‐B on human skin cancer cells was achieved through the NF‐κB signaling pathway. The multifaceted impact of resveratrol on various signaling pathways enhances its potential as a candidate for tumor prevention and treatment. In the study conducted by Na Wang et al., it was demonstrated that the synergistic anticancer effects of procyanidins in conjunction with resveratrol may be attributed to the inhibition of the AKT and ERK signaling pathways, coupled with the promotion of the NF‐κB signaling pathway (Wang, Gao, et al., 2023).

### 4.8 Resveratrol induces cancer cell autophagy

Cellular autophagy is a self‐protective mechanism developed by eukaryotic cells during their long‐term evolution as a form of programmed cell death. Autophagy regulates cellular processes such as damage, degeneration, senescence, and death in normal tissues, and has a scavenging effect, which helps maintain the homeostasis of the intracellular environment. The downstream regulator of SIRT1 is adenosine‐activated protein kinase (AMPK), and the SIRT1/AMPK signaling pathway is involved in regulating many physiological processes in the body, such as cell autophagy, cell proliferation and differentiation, protein synthesis and degradation, and apoptosis (Zhao et al., 2017). In cancer cells, the role of cellular autophagy is complex and mainly related to the internal environment. Resveratrol induces autophagy in cancer cells by regulating the SIRT1/AMPK pathway, which in turn promotes apoptosis (Elshaer et al., 2018). The above viewpoint has also been confirmed in the research of Song et al. (2023).

### 4.9 Resveratrol inhibits tumor angiogenesis, invasion, and metastasis

Resveratrol inhibits tumor neovascularization, aggressiveness, and distal metastasis, and slows down tumor progression. Tumor growth and metastasis are dependent on angiogenesis. The expression of extracellular VEGF was reduced after resveratrol was applied to estrogen receptor α‐positive and estrogen β‐receptor‐negative breast cancer cells. Moreover, animal xenograft experiments demonstrated its in vivo angiogenesis inhibitory properties (Garvin et al., 2006). Park et al. (2009) found that the inhibitory effect of resveratrol on cancer cell metastasis was produced by inhibiting NF‐κB activation and ICAM‐1 expression, thereby inhibiting tumor cell adhesion to endothelial cells. Resveratrol was found to inhibit the migration and invasion of human metastatic lung cancer A549 and cervical cancer Hela cells by suppressing NF‐κB and AP‐1‐mediated MMP‐9 expression (Kim et al., 2012).

### 4.10 Resveratrol inhibits cyclooxygenase‐2

Cyclooxygenase‐2 (COX‐2) is an inducible enzyme that is overexpressed in response to tissue injury and inflammation. It has been demonstrated that COX‐2 is closely related to tumor development, tumor cardiovascular production, and tumor metastasis. COX‐2 promotes the synthesis of pro‐inflammatory factors, such as prostaglandins, catalyzed by COX enzymes, which can promote the growth and proliferation of malignant tumor cells. In a study by Zykova et al. (2008), resveratrol directly formed a complex with COX‐2 and reduced COX‐2‐mediated synthesis of PGE2. Since nuclear factor κB (NF‐κB) promotes COX‐2 expression, resveratrol inhibits COX‐2 expression by decreasing NK‐κB activity (Elshaer et al., 2018). Resveratrol also induces COX‐2 nuclear accumulation, increases p53‐dependent anti‐proliferative effects, and reduces ubiquitinylated modifier protein‐1 (SUMO‐1) expression, thereby inhibiting the proliferation of cancer cells (Cheng et al., 2018).

### 4.11 Resveratrol increases chemosensitivity

Tumors can become resistant to different chemotherapeutic drugs. Resveratrol has been shown to enhance the sensitivity of gastric cancer cells to chemotherapeutic agents. Resveratrol modulates various cell signaling molecules that modulate NF‐κB and STAT3 signaling pathways to achieve chemosensitization. Ma L et al. conducted an in vitro study of resveratrol in combination with cisplatin (CIS) and fluorouracil for cancer treatment and showed that resveratrol increased the inhibitory effect of fluorouracil on the invasiveness of colon cancer cells (Cosco et al., 2015). Ma et al. (2015) showed that resveratrol exhibited anticancer effects on non‐small cell lung cancer H838 and H520 cell lines and enhanced the antitumor effects of cisplatin by modulating the mitochondrial apoptotic pathway.

## 5 CONCLUSION

Resveratrol exerts its antitumor effects through multiple mechanisms and has great potential as an anticancer agent for the prevention and treatment of many types of cancer. The literature analysis results of this study indicate that a new frontier in the anticancer effects of resveratrol may involve its participation in regulating the cell cycle, modulating cancer genes, blocking cancer cell pathways, inducing autophagy, and promoting apoptosis. These mechanisms may contribute to the therapeutic and preventive roles of resveratrol in cancer.

## AUTHOR CONTRIBUTIONS

**Qiang Fu:** Conceptualization (lead); data curation (lead); formal analysis (lead); methodology (lead); resources (equal); software (lead); supervision (lead); writing – original draft (lead). **Zhongqi Lu:** Conceptualization (equal); data curation (equal); formal analysis (equal); methodology (equal); resources (equal); writing – original draft (equal). **Ying Chang:** Conceptualization (equal); data curation (equal); formal analysis (equal); methodology (equal). **Tiefeng Jin:** Conceptualization (equal); funding acquisition (equal); methodology (equal); validation (equal); visualization (equal); writing – review and editing (equal). **Meihua Zhang:** Conceptualization (equal); resources (equal); supervision (equal); visualization (equal); writing – review and editing (lead).

## FUNDING INFORMATION

This research was financially supported by the National Natural Science Foundation of China (grant number 81960554) and Jilin Provincial Science and Technology Department (grant number YDZJ202201ZYTS179; YDZJ202301ZYTS131).

## CONFLICT OF INTEREST STATEMENT

The authors affirm that this research was conducted without any potential conflicts of interest arising from commercial or financial relationships.

## Data Availability

The data underpinning the findings of this study can be obtained from the corresponding author upon request.
